# Arsenic exposure is associated with DNA hypermethylation of the tumor suppressor gene p16

**DOI:** 10.1186/s12995-014-0042-5

**Published:** 2014-12-20

**Authors:** Guangming Lu, Huiwen Xu, De Chang, Zhenglai Wu, Xiaoyuan Yao, Shiying Zhang, Zhenlong Li, Jieben Bai, Qing Cai, Wen Zhang

**Affiliations:** Institute of Health Management, Chinese PLA General Hospital, Beijing, 100853 China; Institute for Medical Device Standardization Administration, National Institute for Food and Drug Control, Beijing, 100050 China; Department of Respiratory Medicine, General Hospital of Chinese People’s Armed Police Forces, Beijing, 100039 China; School of Basic Medicine, Peking Union Medical College, Beijing, 100005 China; Institute of Environmental Health Monitoring, Chinese Center for Disease Control and Prevention, Beijing, 100021 China; Bameng Hygiene and Epidemic Prevention Station, Inner Mongolia, 015000 China; Hangjinhouqi Hygiene and Epidemic Prevention Station, Inner Mongolia, 015400 China; Key Laboratory of Molecular Biology, Chinese PLA Air Force General Hospital, Beijing, 100036 China

**Keywords:** Arsenic, p16 gene, DNA methylation, Case–control study, Cancer

## Abstract

**Background:**

Occupational and environmental exposure to inorganic arsenic leads to development of cancer and represents a significant health hazard in more than 70 countries. The underlying mechanism for arsenic-induced carcinogenesis remains unclear. Laboratory studies suggest that arsenic is a poor mutagen but may cause epigenetic silencing of key tumor suppressor genes such as p16 through DNA hypermethylation. However, the evidence for an association between human arsenic exposure and abnormal DNA methylation of tumor suppressor genes is lacking.

**Findings:**

Paired case–control studies were conducted involving 40 individuals with high arsenic exposure and arsenicosis, 40 individuals with similarly high exposure to arsenic but without arsenicosis, and 40 individuals with normal exposure to arsenic. DNA methylation status of p16 was determined using methylation-specific PCR. Conditional logistic regression analysis showed that DNA hypermethylation of p16 gene was significantly associated with high arsenic exposure (Odds Ratio = 10.0, P = 0.0019) independently of the development of arsenicosis (Odds Ratio = 2.0, P = 0.1343).

**Conclusions:**

High exposure of arsenic in human is positively linked to DNA hypermethylation of p16 gene, suggesting that epigenetic silencing of key tumor suppressor may be an important mechanism by which arsenic promotes cancer initiation.

**Electronic supplementary material:**

The online version of this article (doi:10.1186/s12995-014-0042-5) contains supplementary material, which is available to authorized users.

## Findings

### Introduction

Inorganic arsenic is a widespread pollutant. Human exposure to arsenic can occur occupationally and environmentally. In many Asian developing countries, synthetic arsenates are commonly used as agricultural insecticides and poisons. In addition, contamination of arsenic in drinking water and through improper coal burning and disposal represents a major health concern, affecting over 137 million people in more than 70 countries [1]. Chronic arsenic exposure is associated with a range of adverse health outcomes, including skin lesions (hyperpigmentation, keratosis), neuropathy and cardiovascular diseases [2,3]. In particular, high levels of arsenic exposure are associated with increased risks of cancers in skin, liver, kidney and bladder [4,5]. However, the mechanism(s) underlying arsenic’s carcinogenic potential remain elusive.

Laboratory investigations using cell culture and animal models indicate that arsenic is a poor mutagen [6,7], suggesting that genetic mutation may not be critically involved in arsenic-induced tumor development. In contrast, it is well-documented that after entering human body, arsenic is metabolized to monomethylarsonic acid and dimethylarsenic acid and such reactions rely on S-adenosylmethionine (SAM) as methyl donor [8]. SAM is an essential co-factor for cellular methyltransferases including DNA methyltransferases (DNMTs), which are responsible for the generation of 5-methylcytosine in DNA. It was hypothesized that by interfering with SAM metabolism, elevated arsenic levels might lead to abnormal DNA methylation. Indeed, treating cells with arsenic altered the global and local DNA methylation patterns [9-11]. Since DNA methylation at gene promoters is tightly linked to the regulation of gene expression [12], arsenic may facilitate tumor initiation and development through epigenetic modulation of key oncogenes and/or tumor suppressors.

*p16* (also known as CDKN2A) is a well-established tumor suppressor. *p16* serves as an important regulator of cell cycle during G1/S phase progression and represents a critical barriers for cellular transformation [13]. *p16* is implicated in a variety of human cancers including dermatological malignancies [14]. Importantly, in addition to genetic deletion and mutation, *p16* is frequently silenced in human tumors through DNA hypermethylation [15]. As an attempt to understand the mechanistic link between arsenic exposure and increased cancer risk, we determined whether high exposure to arsenic in human is associated with altered DNA methylation of *p16* gene.

## Results

We conducted a paired case–control study approved by the Chinese PLA General Hospital review board. 40 case subjects were recruited from villages in Bameng, Inner Mongola of China where the drinking water is contaminated with high levels of arsenic (>0.05 mg/L, average = 0.6 mg/L). The selection criteria include a clear drinking history and a positive diagnosis of arsenic poisoning (arsenicosis). Two cohorts of control subjects, matched with respect to age, sex, ethnicity and socioeconomical status, were recruited including: 1) individuals from the same villages with high arsenic exposure but showed no clinical signs of arsenicosis; and 2) individuals from the neighboring villages where the drinking water is not contaminated with arsenic (<0.05 mg/L, average = 0.02 mg/L). Table 1 summarizes the descriptive characteristics of the study participants. None of the parameters is statistically significantly different between case and control groups.

**Table 1:** **Descriptive characteristics of study subjects**

**Parameters**	**Individuals with High arsenic exposure and arsenicosis**	**Individuals with High arsenic exposure and without arsenicosis**	**Individuals with low arsenic exposure**
Total Subjects	40	40	40
Sex (Male/Female)	27/13	27/13	27/13
Age (mean ± SD years)	44.125 ± 12.015	44.250 ± 11.931	43.650 ± 12.223
Ethnicity (% of Han)	97.5	100	100
Occupation (% of farmers)	92.5	97.5	90

Interviews were conducted between trained practitioners and study subjects with a structured epidemiological questionnaire including details about individual’s demographic factors, drinking water history and condition, smoking index, alcohol consumption, past medical history and current illness. 3 ml of vein blood were drawn from study subjects. Whole blood leukocytes were isolated through centrifugation and stored at −20°C. DNA was extracted for methylation-specific (MS) PCR reactions to determine the DNA methylation levels of *p16* gene as described previously [16]. In brief, each sample was treated with bisulphite to convert unmethylated but not methylated cytosine to uracil. The samples were then amplified with two sets of primers targeting *p16* promoter regions, one for unmethylated DNA (Forward 5′-TTATTAGAGGGTGGGGTGGATTGT-3′; Reverse 5′-CAACCCCAAACCACAACCATAA-3′) and one for methylated DNA (Forward 5′- TTATTAGAGGGTGGGGCGGATCGC-3′; Reverse 5′- GACCCCGAACCGCGACCGTAA-3′). The PCR products were analyzed by polyacrylamide gel electrophoresis and ethidium bromide staining. The appearance of a band of ~150 bp indicates the presence of *p16* methylation in the blood. A representative result of the MS-PCR assay with positive and negative controls is shown in Additional file 1: Figure S1.

As shown in Table 2, 26 of 40 (65%) subjects in high arsenic exposure with arsenicosis group had *p16* hypermethylation, while 19 of 40 (47.5%) subjects in high arsenic exposure without arsenicosis group were *p16* methylation positive. In sharp contrast, only 9 of 40 (20%) subjects in low arsenic exposure group were *p16* methylation positive (P < 0.01, Fisher’s Exact test). These results suggest that there is a significant correlation between *p16* hypermethylation and arsenic exposure independent of the development of arsenicosis.

**Table 2:** **Frequencies of** ***p16*** **methylation in study groups**

**Group**	***p16*** **methylation positive**	***p16*** **methylation negative**	**Total**
High arsenic exposure and arsenicosis	26 (65%)	14	40
High arsenic exposure and without arsenicosis	19 (47.5%)	21	40
Low arsenic exposure	8 (20%)**	32	40

**P < 0.01.

To extend our analysis, variables collected through interviews and MS-PCR results were assigned with quantified value as shown in Additional file 2: Table S1. Crude odds ratios (OR) and estimates of relative risk were calculated by univariate analysis. To identify variables that were independently associated with arsenic exposure, multivariate analyses were performed using conditional logistic regression methods. All P-values resulted from two-sided statistical tests. The FREQ and PHREG procedures in Statistical Package SAS 6.12 were employed.

We first compared case subjects (high exposure to arsenic with arsenicosis) to control subjects with minimal exposure to arsenic. 12 variables were included in the univariate logistic regression analysis and Table 3 summarizes the results of five of them. DNA hypermethylation of *p16* showed a highly significant association with case subjects (OR = 10.0,P = 0.0019), while none of the other variables reached statistical significance.

**Table 3:** **Logistic regression analysis results comparing cases to controls with low exposure to arsenic**

**Variables**	**Regression coefficient**	**Standard error**	**χ** ^**2**^	**P-value**	**Odds ratio**
Education	0.000000	0.57735	0.00000	1.0000	1.000
Years of drinking water	1.302854	0.76139	2.92805	0.0871	3.680
Smoking Index	0.017927	0.02852	0.39504	0.5297	1.018
Alcohol Consumption	0.677061	0.48951	1.91309	0.1666	1.968
*p16* methylation	2.302585	0.74162	9.63981	0.0019	10.000**

**P < 0.01.

We next compared case subjects to control subjects who are exposed to high arsenic levels without arsenicosis. 13 variables were included in the univariate logistic regression analysis and Table 4 summarizes the results of six of them. The only variables displaying significant association with case subjects were years of drinking contaminated water (OR = 1.192, P = 0.0154) and arsenic concentration in drinking water (OR = 4.2,P = 0.0039). None of the other variables, including *p16* DNA methylation (OR = 2, P > 0.05), reached statistical significance.

**Table 4:** **Logistic regression analysis results comparing cases to controls with high exposure to arsenic without arsenicosis**

**Variables**	**Regression coefficient**	**Standard error**	**χ** ^**2**^	**P-value**	**Odds ratio**
Education	0.470004	0.57009	0.67970	0.4097	1.600
Years of drinking water	0.175220	0.07234	5.86663	0.0154	1.192*
Smoking Index	0.031609	0.02639	1.43463	0.2310	1.032
Alcohol Consumption	−1.323988	0.68202	3.76851	0.0522	0.266
Arsenic Concentration in water	1.435085	0.49761	8.31708	0.0039	4.200**
*p16* methylation	0.693147	0.46291	2.24211	0.1343	2.000

*P < 0.05; **P < 0.01.

Collectively our analysis results suggest that *p16* DNA hypermethylation is significantly associated with high arsenic exposure and such association is independent of whether the subjects develop arsenicosis. On the other hand, the water drinking history as well as the amount of arsenic in the drinking water represent critical risk factors for arsenic poisoning.

## Discussion

Despite significant efforts to eliminate arsenic from industrial processing and agricultural use, occupational and environmental exposures to toxic levels of arsenic are still common in developing countries. Therefore understanding the link between arsenic intake and adverse health effects such as cancer will exert significant public health benefit. The carcinogenic mechanism(s) of arsenic have been extensively studied in laboratories using animal and cell culture models. Intriguingly, in contrast to most chemical carcinogens, arsenic only weakly induces genetic mutation [7]. On the other hand, multiple groups have reported that arsenic can induce significant changes in DNA cytosine methylation. For example, Zhao et al. reported that chronic arsenic treatment in rat liver epithelial cells induced malignant transformation which was accompanied by global DNA hypomethylation [10]. Mass and Wang showed that exposing human lung A549 cells to sodium arsenite produced significant dose-responsive hypermethylation at the promoter of *p53* tumor suppressor gene [9]. Furthermore, recently Cui et al. demonstrated that giving mice arsenic in the drinking water induced formation of lung adenocarcinoma with DNA hypermethylation of *p16* [11]. In the current study, we present data that human exposed to high levels of arsenic are significantly more likely to have *p16* DNA hypermethylation. Our findings as well as others’ support the hypothesis that arsenic may act as a potent “epi-mutagen” to epigenetically modify key tumor suppressor genes. As DNA methylation at promoters can silence gene expression in a heritable manner, this could be a critical mechanism by which cells exposed to arsenic overcome barriers to malignant transformation.

Previously Chanda et al. examined human subjects exposed to arsenic-contaminated drinking water in West Bengal in India and found that DNA hypermethylation of *p53* and *p16* positively correlated with arsenic levels in drinking water [17]. Studies of individuals using arsenic-rich coal with indoor unventilated stoves in Guangzhou, China also found trends of increased *p16* methylation and reduced protein expression [18]. Our results are consistent with these findings. In addition, we for the first time showed that *p16* DNA methylation levels are not significantly different between subjects with high exposure to arsenic and arsenicosis and subjects with similar arsenic exposure without arsenicosis. This result excludes the possibility that *p16* DNA hypermethylation is a secondary pathological consequence of arsenic poisoning. Furthermore, the result suggests that *p16* hypermethylation precedes the development of clinical symptoms and may be an important risk factor for arsenic-related diseases such as cancer. This is consistent with clinical observations that loss of *p16* through genetic or epigenetic inactivation is an early event in tumor progression [19]. A weakness of our study was the use of whole blood leukocytes for *p16* methylation analysis. Although this approach has been successful to study disease-related epigenetic changes [20], samples from more relevant tissue types such as skin would potentially provide more information.

There are several limitations of current study. First, although the high sensitivity of MS-PCR assay allows us to measure *p16* methylation with a limited amount of human blood sample, this assay is qualitative rather than quantitative. Future study with bisulfite sequencing will be required to validate our findings and to provide a quantitative assessment of the correlation between arsenic exposure and *p16* methylation. Second, because our sampling sites are located in remote areas with no laboratory access which makes the preservation of RNA from blood very challenging, our study did not provide a measurement of p16 RNA and protein expression. Although the association between *p16* promoter methylation and gene repression has been firmly established [21,22], it would be important in the future to demonstrate that the epigenetic silencing of *p16* affects its expression. In conclusion, our study demonstrates that arsenic exposure is associated with *p16* DNA hypermethylation in human, which may be an important risk factor for arsenic-induced tumor development [23,24]. Further investigations are needed to reveal how arsenic metabolism specifically alters cellular epigenetic state. As clinically approved drugs targeting DNMTs are available, our results suggest that pharmacological reversal of DNA hypermethylation at tumor suppressor genes may offer therapeutic benefits to arsenic exposed individuals.

## Consent

Written informed consent was obtained from the patient for the publication of this report and any accompanying images.

## Additional files

Additional file 1: Figure S1. Representative result of MS-PCR assay. Additional file 2: Table S2. Quantification of variables for logistic regression analysis.

## Abbreviations

SAM: S-adenosylmethionine
DNMTs: DNA methyltransferases
MS-PCR: Methylation-specific PCR
