# Supplementary material for: Arsenic exposure is associated with DNA hypermethylation of the tumor suppressor gene p16
Source: J Occup Med Toxicol. 2014 Dec 20;9:42. doi: 10.1186/s12995-014-0042-5 (PMC4297462; doi:10.1186/s12995-014-0042-5)
Supplement: Additional file 1: Figure S1. — Representative result of MS-PCR assay. [file 12995_2014_42_MOESM1_ESM.docx]

**Additional file 1:** **Figure 1**

**
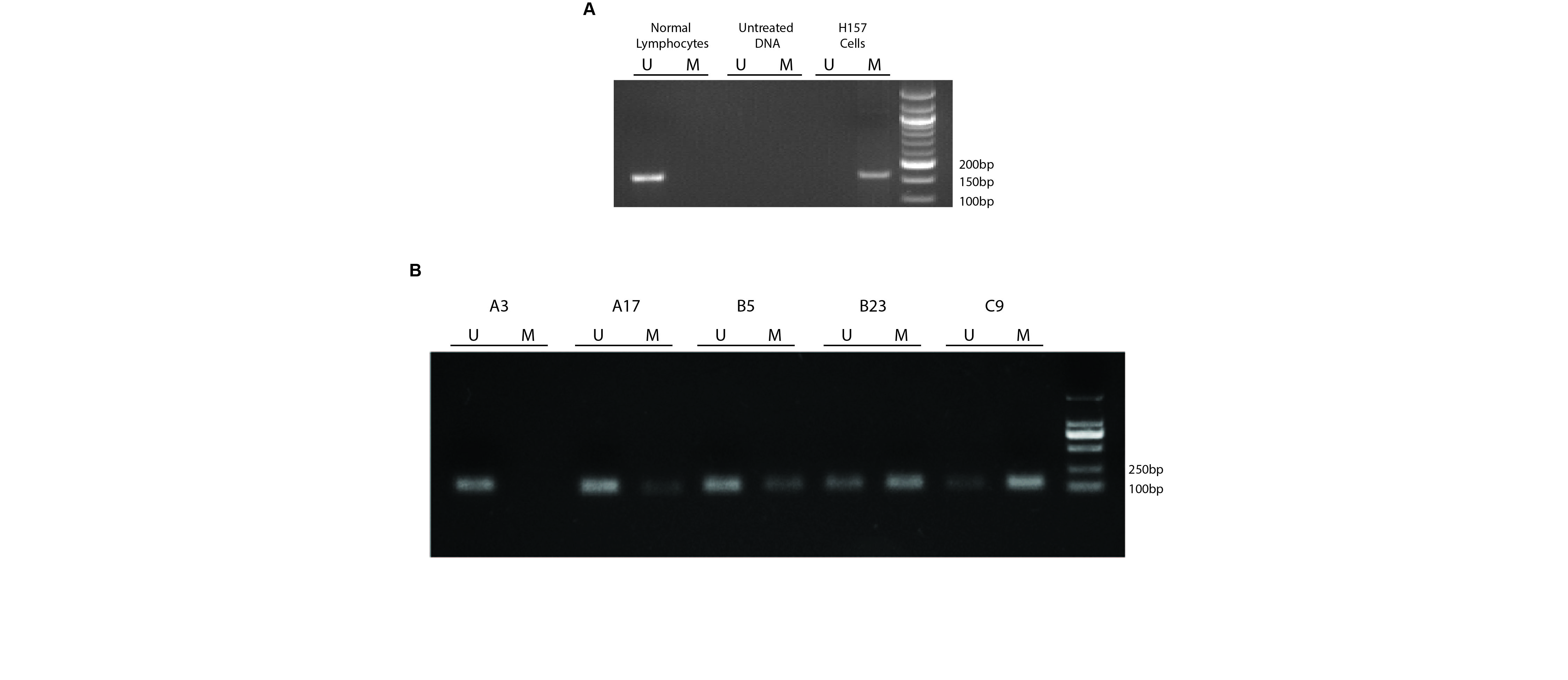
Additional file 1: Figure 1: Representative result of MS-PCR assay.**

(A) Image of the gel electrophoresis analysis of PCR products from MS-PCR assays. DNA extracted from bisulfite-treated DNA from normal lymphocytes (negative control), H157 lung cancer cell line (*p16*-methylation positive control), and untreated DNA from H157 lung cancer cell line (negative technical control) was amplified by primer sets targeting unmethylated (U) or methylated (M) *p16* promoter regions. The appearance of a band of ~150 bp with M-primers indicates the presence of *p16* methylation.

(B) A representative image of the gel electrophoresis analysis of PCR products from MS-PCR assays. DNA extracted from individuals was amplified by primer sets targeting unmethylated (U) or methylated (M) *p16* promoter regions. The appearance of a band of ~150 bp with M-primers indicates the presence of *p16* methylation in the blood. Samples shown are selected from low arsenic exposure group (A3 and A17); high arsenic exposure without arsenicosis group (B5 and B23); and high arsenic exposure with arsenicosis group (C9).
