# Supplementary material for: Arsenic exposure is associated with DNA hypermethylation of the tumor suppressor gene p16
Source: J Occup Med Toxicol. 2014 Dec 20;9:42. doi: 10.1186/s12995-014-0042-5 (PMC4297462; doi:10.1186/s12995-014-0042-5)
Supplement: Additional file 2: Table S2. — Quantification of variables for logistic regression analysis. [file 12995_2014_42_MOESM2_ESM.docx]

| Variables | Quantification Criteria |  | Variables | Quantification Criteria |
| --- | --- | --- | --- | --- |
| Gender | Male (1)  Female (0) |  | Lung Cancer | Yes (1)  No (0) |
| Profession | Farmer (1)  Otherwise (0) |  | Pneumonia | Yes (1)  No (0) |
| Race | Han (1)  Otherwise (0) |  | Heart Disease | Yes (1)  No (0) |
| Education | Middle School & Above (1)  Otherwise (0) |  | Years of drinking | <5 (0)  ≥5 (1) |
| Alcohol Consumption | No (0)  <100 g/day (1)  >= 100 g/day (2) |  | Smoking Index | Daily amount x Years smoking / Age at first smoke |
| Skin Diseases | Yes (1)  No (0) |  | *p16* methylation | Yes (1)  No (0) |

**Additional file 2: Table 1 Quantification of variables for logistic regression analysis**
